# Radiological and Laboratory Findings of Patients with COVID-19 Infection at the Time of Admission

**DOI:** 10.30699/IJP.2020.128909.2415

**Published:** 2020-10-21

**Authors:** Saeed Mirsadraee, Mihan Pourabrollah Toutkaboni, Mehrdad Bakhshayeshkaram, Mitrasadat Rezaei, Elham Askari, Sara Haseli, Nazanin Sadraee

**Affiliations:** 1 *Royal Brompton and Harefield NHS Foundation Trust, Sydney St, London, UK. *; 2 *Chronic Respiratory Diseases Research Center, National Research Institute of Tuberculosis and Lung Diseases (NRITLD), Shahid Beheshti University of Medical Sciences, Tehran, Iran *; 3 *Virology Research Center, National Research Institute of Tuberculosis and Lung Diseases (NRITLD), Shahid Beheshti University of Medical Sciences, Tehran, Iran*; 4 *Medical Imaging Research Center, Department of Radiology, Shiraz University of Medical Sciences, Shiraz, Iran*

**Keywords:** COVID-19, Radiology, Laboratory findings, CT scan, Chest X-ray

## Abstract

**Background & Objective:**

Diagnosis of coronavirus disease 2019 (COVID-19) can be challenging, especially when the real-time quantitative reverse transcription polymerase chain reaction (RT-PCR) is not available or it is negative. In this study, we evaluated imaging and laboratory findings in a group of patients with a multidisciplinary diagnosis of COVID-19 pneumonia.

**Methods:**

A total of 163 patients with a clinical diagnosis of COVID-19 pneumonia admitted to a specialised respiratory centre in Tehran, Iran were enrolled in this study. The distribution and characteristics of presenting radiological and laboratory findings were evaluated and the relationship to the outcome was investigated.

**Results:**

RT­PCR was positive in 92 patients. The diagnosis of COVID-19 in RT-PCR negative patients was made on clinical and radiological features (n=71) and 24 (14.7%) patients died of disease. The common computed tomography (CT) scan findings included ground-glass (94%) and consolidating opacification (12%), mainly in the lower lobes (90%). Peripheral and central lung changes were observed in 90% and 52% of patients, respectively. Lymphopenia, positive CRP, and raised LDH were present in 32%, 65%, and 96% of cases, respectively. A raised LDH of >500U/L was the best predictor of death in these patients (R^2^=0.6623; OR=24.4). Other markers of outcome included male gender, age (>50 years), lymphopenia, and severe CXR changes.

**Conclusion:**

Diagnosis of COVID-19 can be challenging, and a multidisciplinary approach is often needed. Whilst RT-PCR is still the standard diagnostic test, a negative test should be interpreted with caution. Blood tests and imaging can be useful in the diagnosis, monitoring, and risk assessment in patients with COVID-19.

## Introduction

Since the identification of a novel human coronavirus disease (COVID-19) in late December 2019 as the cause of viral pneumonia in Wuhan, the disease has been rapidly spreading outside of China. In Iran, the first confirmed case was reported on 19 February 2020; since then, the disease has evoked a pandemic in many regions of the country. The pandemic has caused significant pressure on the clinical services due to the sheer number of patients needing a high level of clinical and respiratory support. During an outbreak, the provision of diagnostic services can be challenging due to extremely high demand, availability, and safety. 

Diagnosis of COVID-19 is based on clinical history, and a favourable laboratory and/or radiological tests. Patients with COVID-19 commonly show symptoms of fever and cough (>80%) and less commonly with shortness of breath (31%), muscle ache (11%), and confusion (9%) (1). A study of 1,014 patients from Wuhan, China, reported a positive rate of 59% for RT-PCR assay and 88% for thoracic CT in patients suspected of COVID-19. Whilst CT was positive in 97% of cases with positive RT-PCR, CT diagnosis of COVID-19 was considered highly likely in 48%, and probable in 33% of cases with negative RT-PCR (2). 

While previous studies described laboratory and imaging changes in COVID-19 pneumonia, the relationship between the changes and the outcome is still the subject of a hot debate. This study describes the pattern of laboratory and radiological results and their relationship to the outcome (death) in a series of hospitalised COVID-19 patients in Tehran, Iran.

## Materials and Methods

The institutional ethical board of Masih Daneshvari Hospital (Tehran, Iran) approved this retrospective study and the requirement for informed patient consent was waived (ref. IR.SBMU.NRITLD.REC.1399.019). Data on 170 patients admitted to Masih Daneshvari hospital with clinical or laboratory diagnosis of COVID-19 from February 20^th^ to March 10^th^, 2020 were retrospectively collected and analysed. In addition, seven patients with no imaging records were excluded. The first group of patients had a confirmed diagnosis of COVID-19 infection by RT­PCR (n=92). The second group (n=71) had negative RT-PCR and the diagnosis of COVID-19 infection was based on clinical and radiological presentations.

**Laboratory Tests**

All laboratory results were collected using hospital electronic records. RT-PCR was performed on nasopharyngeal samples, which precisely describe the characteristics of the diagnostic kit (3). In summary, total RNA was extracted using High Pure RNA Isolation (Roche Diagnostics, Penzberg, Germany). RT-PCR for coronavirus genes was performed with Taqman® Premix TAKARA (TaKaRa, Dalian, China) according to the manufacturer’s recommended protocol. Other collected laboratory data included platelet, lymphocyte and neutrophil counts, serum urea and creatinine, C-reactive protein (CRP), erythrocyte sedimentation rate (ESR), aspartate aminotransferase (AST), alanine aminotransferase (ALT), albumin level, and lactate dehydrogenase (LDH).

**Radiological Image Acquisition and Interpretation**

All patients underwent either a chest x-ray (CXR) or an enhanced thoracic computed tomography (CT), or both. When a patient had multiple CXRs, CTs, or laboratory data, the first imaging or laboratory data were considered. 

All CT images were obtained using a 16 slice Brilliance CT scanner (Philips medical system, Cleveland, OH) in a supine position. The scanning parameters included: tube voltage: 100-120 kVp; tube current with modulation (50-100 mAs); pitch: 0.8-1.5; matrix: 512x512; slice thickness: 5mm,; and field of view: 350 x350 mm. 

Two board-certified thoracic radiologists (MB and SH) reviewed the images taken on admission and scored CXRs and CT scans by consensus. The CXR and CT images were examined on separate sessions to avoid observational bias. An imaging score was given to the predominant imaging findings in each zone on CXR (upper, middle, and left in each lung) and each lobe on CT (3 lobes in right and 2 lobes in the left lung). On CXR, a patchy opacification was scored 1, and a confluent consolidation was scored 2. On CT, ground glass changes (including crazy paving) of <3cm and >3cm were scored 1 and 2, respectively. A dense consolidation on CT was scored 3. The maximum total score on CXR and CT was 12 and 15, respectively. A CXR score of 0, 1-6, and 7-12 was defined as normal, less than severe, and severe, respectively. A CT score of 0, 1-7, and 8-15 was defined as normal, less than severe, and severe, respectively.

**Statistical Analysis**

Statistical analyses were performed using the SAS software (Statistical Analysis System (2003) v9.13 (SAS Institute Inc, Cary, North Carolina, USA). The dependent variable was survival outcome status (death or no-death). The independent data consisted of several continuous and categorical variables, including gender, age groups (under and above 50 or 60 years), RT-PCR results (positive or negative), blood results, and imaging scores (normal, less than severe and severe). The association between the independent variables and the outcome was analysed using analysis of variance (ANOVA) and Chi-squared test. A p-value of <0.05 was defined as significant. 

## Results

Patients’ demographics and laboratory charac-teristics are summarised in Table 1. The study population comprised 163 cases (116 (71%) males and 47 (39%) females). The mean age was 51 years (SD: 16, range: 2-87). Of these cases, 92 (56%) had a positive RT-PCR test and 71 (44%) were RT-PCR negative at presentation. Twenty patients (13%) were admitted to the intensive care unit (ICU). Twenty-four patients died (14.7%), of whom 19 were admitted to the ICU. The mean age for patients who died was 60 years (median: 64; 21-83). The mean age for survivors was 49 years (median: 50; 2-87). Four out of 24 (16.7%) patients who died were female.

On the admission CT (Table 2), the common findings included: patchy ground-glass change (94%), confluent consolidation (12%), and crazy paving (3%). The airway changes were relatively rare. Whilst over 90% of patients had peripheral lung lesions, only 52% of patients had central lung changes. In more than 90% of patients, there were lesions in the lower lung lobes. Lesions were more commonly present in the right upper lobe than the left upper lobe or middle lobes. There was no evidence of background emphysema, and 3 patients (9%) had evidence of interstitial lung disease. While only 2 patients (4%) with positive RT-PCR results had normal CT scans, 12 (23%) with normal CT scans presented with negative RT-PCR. The latter patients developed the typical findings of COVID-19 on the follow-up imaging. The diagnosis of COVID-19 in the latter was based on the typical clinical presentation of acute respiratory distress.

**Table 1:** Clinical characteristics and laboratory findings in patients admitted with COVID-19 pneumonia

	All patients (n=163)	+ve RT-PCR (n=92)	-ve PCR (n=71)
Demographics			
Age (years)	51 (2-87, SD=16)	52 (3-87, SD=16)	49 (2-83, SD=17)
**<30**	20 (12%)	10 (11%)	10 (14%)
**30-49**	52 (32%)	24 (26%)	28 (39%)
**50-69**	73 (45%)	47 (51%)	26 (37%)
**≥70**	18 (11%)	11 (12%)	7 (10%)
Gender			
**M**	116 (71%)	67 (73%)	49 (69%)
**F**	47 (29%)	25 (27%)	22 (31%)

Laboratory results			
White blood cell count (/μl)	7471 (1700-25000, SD=3775)	6953 (2200-25000, SD=811)	8535 (1700-18790, SD=1388)
**>11000**	26 (16%)	7 (8%)	19 (27%)
**4000-11000**	114 (72%)	72 (80%)	42 (61%)
**<4000**	19 (12%)	11 (12%)	8 (12%)
Lymphocyte count (/μl)	1439 (range, SD=830)	1479 (348-4531, SD=180)	1291 (420-6044, SD=263)
**≥ 1500**	55 (35%)	NA	55 (83%)
**>** **1000-<1500**	52 (33%)	41 (45%)	11 (17%)
**<1000**	51 (32%)	51 (55%)	NA
AST (IU/Lit)	43 (14-251, SD=34)	43 (14-251, SD=8)	46 (16-230, SD=11)
≤30	60 (39%)	29 (33%)	31 (46%)
30-100	86 (56%)	52 (60%)	34 (51%)
≥100	8 (5%)	6 (7%)	2 (3%)
ALT (IU/Lit)	39 (8-316, SD=44)	41 (8-227, SD=10)	50 (8-316, SD=15)
**≤40**	116 (75%)	92 (100%)	23 (38%)
**40-100**	31 (20%)	NA	31 (51%)
**≥100**	7 (5%)	NA	7 (11%)
LDH (U/L)	510 (183-2954, SD=305)	599 (183-2954, SD=63)	534 (238-882, SD=89)
**≤250**	5 (4%)	4 (5%)	1 (2%)
**250-500**	69 (57%)	54 (67%)	15 (38%)
**500-1000**	44 (37%)	22 (27%)	22 (55%)
**≥1000 U/Lit**	3 (2%)	1 (1%)	2 (5%)
Albumin (g/dl)	3.3 (1.7-4.8, SD=0.5)	3.4 (2.2-4.3, SD=0.1)	3.2 (1.7-4.7, SD=0.2)
**< 2.8**	12 (14%)	4 (8%)	8 (22%)
**2.8- 3.8**	59 (69%)	37 (76%)	22 (59%)
**≥ 3.8**	15 (17%)	8 (16%)	7 (19%)
Platelet (x10^3^/μl)	192 (10-528, SD=90)	195 (35-528, SD=19)	193 (10-516, SD=28)
**≥150**	107 (67%)	55 (61%)	52 (75%)
**<150**	52 (33%)	35 (39%)	17 (25%)
CRP ≤10 mg/L **(Negative)**	45 (35%)	20 (28%)	25 (45%)
CRP >10 mg/L **(Positive)**	82 (65%)	51 (72%)	31 (55%)
ESR (mm/hour)	42 (2-125, SD=32)	49 (2-125, SD=6)	46 (2-119, SD=6)
**≤20 mm/hour (M)** **≤30 mm/hour (F)**	44 (35%)	28 (39%)	16 (30%)
**20-100 mm/hour (M)** **30-100 mm/hour (F)**	73 (59%)	38 (54%)	35 (65%)
**≥100 mm/hour**	8 (6%)	5 (7%)	3 (5%)
Creatinine (mg/dl)	1.26 (0.4-12.2, SD=1.00)	1.24 (0.5-12.2, SD=0.23)	1.33 (0.4-6.2, SD=0.33)
**≤1.3 mg/dl (M)** **≤1.1 mg/dl(F)**	125 (77%)	70 (76%)	55 (77%)
**>1.3 mg/dl (M)** **>1.1 mg/dl (F)**	38 (23 %)	22 (24%)	16 (23%)
Urea (mg/dl)	37 (10-158, SD=24)	35 (100-152, SD=5)	52 (11-158, SD=7)
**≤50**	143 (88%)	82 (89%)	61 (86%)
**>50**	20 (12%)	10 (11%)	10 (14%)

**Table 2:** CT characteristics of COVID-19 pneumonia at presentation

		All patients (n=33)	+ve RT-PCR (n=20)	-ve PRT-CR (n=13)
CT scan				
Distribution	RUL	27 (81.9%)	17 (85%)	10 (77%)
	RML	20 (60%)	14 (70%)	6 (43%)
	RLL	30 (91%)	19 (95%)	11 (84%)
	LUL	22 (67%)	15 (75%)	7 (54%)
	LLL	30 (91%)	18 (90%)	12 (92%)
			
	Peripheral	31 (94%)	18 (90%)	13 (100%)
	Central	17 (52%)	12 (60%)	5 (38%)
	Anterior	25 (76%)	19 (95%)	6 (43%)
	Posterior	32 (97%)	19 (95%)	13 (100%)
Characteristics	GG opacification	31 (94%)	20(100%)	11 (85%)
	Consolidation	4 (12%)	1 (5%)	3 (23%)
	Crazy paving	1 (3%)	1 (5%)	0
	Tree-in-bud	1 (3%)	1 (5%)	0
	Airway impaction	1 (3%)	1 (5%)	0
	Bronchial wall thickening	4 (12%)	4 (20%)	0
Associated lung disease	Interstitial lung disease	3 (9%)	3 (15%)	0

**Fig. 1:**
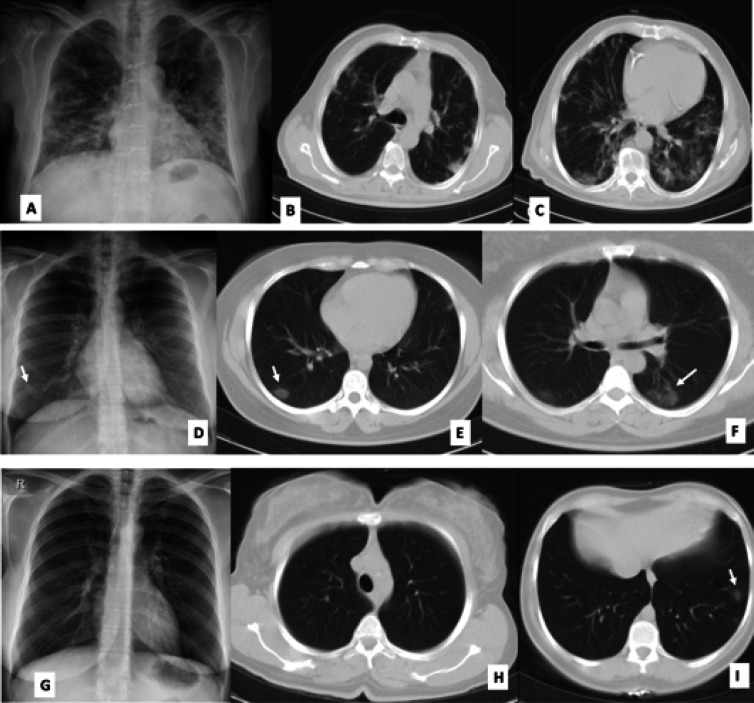
Variety of chest graph changes in patients with confirmed COVID-19 pneumonia. In patient 1, radiograph shows multifocal patchy airspace opacifications in both lungs, more severe in the mid and lower zones of the lungs (A; arrows). CT shows peripheral ground-glass opacities and consolidations in the upper and lower lobes (B&C). In the second patient, radiograph (D) shows a subtle opacity in the right lower zone corresponding to peripheral ground-glass opacity in the right lower lobe (arrow; E). The opacification in the left lower lobe (F) is not visible on the radiograph. In patient 3 (G) the radiograph was normal and missed small opacity in the left lower lobe (I)

Among the patients who had both CXR and CT (n=46) on admission, 14 (30%) had normal CXR and CT, 3 (7%) had normal CXR but abnormal CT, and 29 (63%) had abnormal CXR and CT. There was no case with an abnormal CXR but a normal CT. Based on the above, the sensitivity and specificity of CXR compared to CT were 90.6% and 100%, respectively (Figure 1). 

In cases with positive RT-PCR, 82 patients underwent CXR, of whom 31 (38%) presented with normal CXR, and 27 (33%) and 24 (29%) showed less than severe and severe changes, respectively. In 88% of patients with abnormal CXR, the changes were bilateral. The CXR findings were present in 98%, 80%, and 47% of cases in the lower, mid, and upper zones of the lungs, respectively. Patchy opacifications were found in 73% of the cases and dense consolidations were seen in 88% of the cases. There was no pleural effusion or pneumothorax on CT or CXR. 

Laboratory results are summarised in Table 1. The majority (72%) of patients had normal white blood cell count, 12% had leukopenia, and 16% had leukocytosis. Approximately one third of patients showed marked lymphopenia (<1000/μL). CRP and ESR levels were raised in 65% of patients. Thrombocytopenia was detected in one third of patients. The majority of patients demonstrated raised levels of LDH, and a significant increase in LDH levels (>500U/L) was present in 39% of patients. Abnormal AST and ALT levels were observed in 61% and 25% of patients, respectively. While most patients had hypo-albuminaemia, a severe reduction in albumin levels was only present in 14% of patients. Most patients (77%) presented with abnormal creatinine levels.

The following variables were associated with a higher risk of death (Chi-squared p-value <0.05): male gender (OR=3.2), age >50 years (OR=4.8), LDH more than 500 (U/L) (OR=24.4), lymphocyte count <1000/μL (OR=3.7), and CXR score >7 (OR=14.6). Other variables including the CT score, blood transaminases, albumin level, CRP, and leukocyte count did not significantly predict the risk of death.

## Discussion

Accurate assessment of patients during the COVID-19 pandemic is vital; but it has been a challenge due to the sheer volume of patients and the limited availability and accuracy of tests. RT-PCR is currently the reference test for the diagnosis of new COVID-19 infection and careers. However, the test has limited sensitivity (<70%) and availability (2, 4, 5). The sensitivity and specificity of RT-PCR test depend on many factors, including the sampling techniques and optimal sample timing that would depend on viral shedding rate (6). CT imaging is reported to be sensitive in the diagnosis of COVID-19 and may be considered in highly suspected cases when RT-PCR is negative or unavailable (2, 5). The reported mean interval between an initial false-negative to positive RT-PCR in patients with COVID-19 is 5.1 days ± 1.5 (2). Instead, CT features of COVID-19 is found in 93-98% of these patients at presentation (2, 5). 

We did not repeat RT-PCR in patients with an initial negative test results and the diagnosis was made on typical clinical and radiological grounds. The published data also suggest that in the correct clinical setting, radiological findings (bilateral ground-glass opacities or consolidations) should prompt a possible diagnosis of COVID-19. Furthermore, a normal chest CT scan would not exclude the diagnosis (7). We observed normal CT at presentation in a small number of cases with positive or negative RT-PCR, but still a diagnosis of COVID-19 was made. All these patients showed radiological signs of COVID-19 later in the course of the disease. 

A study of 81 patients with confirmed COVID-19 by RT-PCR reported that the predominant pattern of abnormalities on CT were bilateral (79%), peripheral (54%), ill-defined (81%), and ground-glass opacification (65%), mainly involving the right lower lobes (8). We observed a similar distribution and airspace abnormalities as described above. In our study, the characteristics of CT findings were similar in the negative and positive RT-PCR groups. It is appreciated that the specificity of CT is limited due to the overlapping features of COVID-19 with other viral pneumonias; however, observation of typical CT features and clinical presentation would support the diagnosis during the pandemic. Repeating RT-PCR and/or imaging should be considered if the diagnosis remains uncertain.

The role of CT imaging in monitoring clinical recovery in patients with COVID-19 is uncertain. Ai *et al.* reported that 42% of patients showed improvement in CT signs before RT-PCR turned negative, but no prognostic value was demonstrated (2). Repetition of imaging should be considered in patients with an unsatisfactory response to treatment to monitor disease progression, and/or complications. CXR is a low-cost investigation and is readily available at the bedside and should be considered for monitoring disease progress and response to treatments, and for the assessment of lines and tubes. Transporting mechanically ventilated infected patients to the imaging department is challenging and limits the availability of the scanner to other patients. 

Variables that were associated with death included a raised LDH, lymphopenia, higher severity of changes on CXR (score>7), male gender, and older age. Our findings showed that 30% of patients older than 70 years of age died. The mortality rate in the <50 and 50-69 year age groups was <6% and 19%, respectively. Other authors also reported that lymphopenia and elevated levels of serum LDH were more common in severe COVID-19 (9). Our data, for the first time, suggested that LDH >500 (U/L) was the best predictor of death with an OR of 24. An R^2^ of 0.6623 by regression analysis indicates that a raised serum LDH explains over 65% of the outcome variable. An increase in LDH levels can be seen in a variety of benign and malignant conditions (10), and in patients with severe COVID-19 pneumonia it appears to be the product of tissue damage. In this cohort, most patients showed increases in LDH level whilst a significant raise was only seen in 39% of cases. Moreover, one third of our patients demonstrated marked lymphopenia. The reported rates of lymphopenia in other COVID-19 studies were 35-83% (1, 11, 12).

While the severity of CXR changes in our study seemed to predict death, the same prediction could not be observed in patients with severe CT changes. This may be related to the timing of CT; in addition, we did not perform serial CT imaging and the changes only reflect a snapshot in time. Pan *et al.* reported an array of CT changes that evolve during 14 days or more follow-up period (13). 

The main limitations of this study included its retrospective design carried out in a single centre. 

## Conclusion

Diagnosis of COVID-19 can be challenging, and a multidisciplinary approach is needed, especially in suspected patients with negative RT-PCR results. Since chest radiographs identified changes of COVID-19 in 70% of patients, those can be considered as the imaging modality of choice for monitoring patients’ progress. A raised LDH of >500U/L was the best predictor of outcome in these patients. Other markers of outcome included male gender, age (>50 years), lymphopenia, and severe CXR changes. 
